# Supplementary material for: Structure of a volume-regulated heteromeric LRRC8A/C channel
Source: Nat Struct Mol Biol. 2022 Dec 15;30(1):52–61. doi: 10.1038/s41594-022-00899-0 (PMC9851909; doi:10.1038/s41594-022-00899-0)
Supplement: Supplementary file 1 — Reporting Summary [file 41594_2022_899_MOESM1_ESM.pdf]

nature research

Corresponding author(s): Raimund Dutzler
Marta Sawicka

Last updated by author(s): Dec 1, 2022

Reporting Summary

Nature Research wishes to improve the reproducibility of the work that we publish. This form provides structure for consistency and transparency in reporting. For further information on Nature Research policies, see our Editorial Policies and the Editorial Policy Checklist.

Statistics

For all statistical analyses, confirm that the following items are present in the figure legend, table legend, main text, or Methods section.

n/a	Confirmed	
☐	☒	The exact sample size (*n*) for each experimental group/condition, given as a discrete number and unit of measurement
☐	☒	A statement on whether measurements were taken from distinct samples or whether the same sample was measured repeatedly
☒	☐	The statistical test(s) used AND whether they are one- or two-sided<br>*Only common tests should be described solely by name; describe more complex techniques in the Methods section.*
☒	☐	A description of all covariates tested
☒	☐	A description of any assumptions or corrections, such as tests of normality and adjustment for multiple comparisons
☐	☒	A full description of the statistical parameters including central tendency (e.g. means) or other basic estimates (e.g. regression coefficient) AND variation (e.g. standard deviation) or associated estimates of uncertainty (e.g. confidence intervals)
☒	☐	For null hypothesis testing, the test statistic (e.g. *F*, *t*, *r*) with confidence intervals, effect sizes, degrees of freedom and *P* value noted<br>*Give P values as exact values whenever suitable.*
☒	☐	For Bayesian analysis, information on the choice of priors and Markov chain Monte Carlo settings
☒	☐	For hierarchical and complex designs, identification of the appropriate level for tests and full reporting of outcomes
☒	☐	Estimates of effect sizes (e.g. Cohen's *d*, Pearson's *r*), indicating how they were calculated

Our web collection on statistics for biologists contains articles on many of the points above.

Software and code

Policy information about availability of computer code

Data collection	Clampex 10.6, EPU 2.9
Data analysis	Clampfit 10.6, Excel 2108, CTFFIND 4.1, RELION 3.1.2 and RELION 4.0-beta, Phenix 1.20.1, Coot 0.9.8, DINO 0.9.6, Chimera 1.15, ChimeraX 1.2.5, HOLE 2.2.005, MSMS, Proteome Discoverer 2.1, Matrix Science 2.7 and 2.7.0.1, Scaffold (Proteome Software Inc. 5.10), XDS, Phaser

For manuscripts utilizing custom algorithms or software that are central to the research but not yet described in published literature, software must be made available to editors and reviewers. We strongly encourage code deposition in a community repository (e.g. GitHub). See the Nature Research guidelines for submitting code & software for further information.

Data

Policy information about availability of data

All manuscripts must include a data availability statement. This statement should provide the following information, where applicable:

- Accession codes, unique identifiers, or web links for publicly available datasets
- A list of figures that have associated raw data
- A description of any restrictions on data availability

The three-dimensional cryo-EM density maps have been deposited in the Electron Microscopy Data Bank under accession numbers EMD-15835 (LRRC8C), EMD-15836 (LRRC8A/C1:1/Sb1), EMD-15837 (LRRC8A/C), EMD-15838 (LRRC8A/C1:3/Sb1), EMD-15839 (LRRC8A/Cendog/Sb1), EMD-15840 (LRRC8ASAM), EMD-15841 (LRRC8ASAM/C). The deposition includes maps of full-length proteins, corresponding both half-maps, the mask used for final FSC calculation as well as relevant higher resolution maps obtained after local refinement. Coordinates have been deposited in the Protein Data Bank under accession numbers 8B40 (LRRC8C), 8B41 (LRRC8A/C1:1/Sb1), 8B42 (LRRC8A/C). Coordinates and structure factors of the X-ray structure of the LRRD of LRRC8C have been deposited in the PDB under accession number 8BEN. The mass spectrometry proteomics data have been deposited to the ProteomeXchange Consortium via the PRIDE (http://

www.ebi.ac.uk/pride) partner repository with the data set identifier PXD035350 and 10.6019/PXD035350. Electrophysiological data that support the findings of this study are provided as source data file.

Field-specific reporting

Please select the one below that is the best fit for your research. If you are not sure, read the appropriate sections before making your selection.

☒ Life sciences ☐ Behavioural & social sciences ☐ Ecological, evolutionary & environmental sciences

For a reference copy of the document with all sections, see nature.com/documents/nr-reporting-summary-flat.pdf

Life sciences study design

All studies must disclose on these points even when the disclosure is negative.

Sample size	No sample size determination was performed. Quantification and functional experiments were performed multiple times with similar results and addition of further data did not change the conclusions of the study. Complete cryo-EM statistics are provided in Table 1 and 2 and in Extended Data Figures 3-4 and 7-9
Data exclusions	In electrophysiology experiments, leaky recordings were discarded. Recordings with no current response were excluded from the analysis. 85% of wild type cells and 35% of LRRC8B,D,E-/- cells showed current response. HEK293 LRRC8-/- cells showed current response in 90% and 70% of patched cells when transfectedwith LRRC8A and LRRC8C or LRRC8ASAM and LRRC8C, respectively.
Replication	Mass spectrometry experiments were replicated as indicated in Extended Data Figure 1 and in the Method section, all replications were successful. Electrophysiology data show the mean of the indicated number of biological replicates, errors are indicated. Recordings were performed multiple times from different transfections. Cells without current response were excluded from the analysis (as described under data exclusion).
Randomization	Randomization is not relevant for this study, as there were no groups allocated in any of the experiments.
Blinding	No blinding was applied as this is deemed not practically feasible.

Reporting for specific materials, systems and methods

We require information from authors about some types of materials, experimental systems and methods used in many studies. Here, indicate whether each material, system or method listed is relevant to your study. If you are not sure if a list item applies to your research, read the appropriate section before selecting a response.

Materials & experimental systems

n/a	Involved in the study	
☐	☒	Antibodies
☐	☒	Eukaryotic cell lines
☒	☐	Palaeontology and archaeology
☒	☐	Animals and other organisms
☒	☐	Human research participants
☒	☐	Clinical data
☒	☐	Dual use research of concern

Methods

n/a	Involved in the study	
☒	☐	ChIP-seq
☒	☐	Flow cytometry
☒	☐	MRI-based neuroimaging

Antibodies

Antibodies used	The antibody used in this study (monoclonal anti-LRRC8A antibody produced in mouse) is commercially available (Sigma, SAB1412855, clone 8H9).
Validation	The mouse anti-LRRC8A antibody (Sigma, SAB1412855) was validated by the supplier and the validation report is available from their website. The antibody was additionally verified by Western Blot using purified LRRC8A protein (Deneka D. et al., Allosteric modulation of LRRC8 channels by targeting their cytoplasmic domains. Nat. Commun., doi: 10.1038/s41467-021-25742-w (2021))

Eukaryotic cell lines

Policy information about cell lines

Cell line source(s)	HEK293S GnTI- (CRL-3022) and HEK293T (CRL-1573) cells were obtained from ATCC. HEK293 LRRC8-/- and HEK293 LRRC8B,D,E-/- cells were obtained from the laboratory of T. J. Jentsch
Authentication	No further authentification was performed for the commercially available celllines. The lack of expression of LRRC8 proteins

Authentication	in HEK293 LRRC8-/- cells was confirmed by electrophysioligy and Western blots with anti-LRRC8A antibody. The expression of LRRC8A proteins in HEK293 LRRC8B,D,E-/- cells was confirmed by Western blots with anti-LRRC8A antibody.
Mycoplasma contamination	The cell lines were tested and are free from mycoplasma contamination.
Commonly misidentified lines (See ICLAC register)	No commonly misidentified lines were used in the study.
